# Chronic traumatic encephalopathy: the dangers of getting "dinged"

**DOI:** 10.1186/2193-1801-1-2

**Published:** 2012-03-12

**Authors:** Shaheen E Lakhan, Annette Kirchgessner

**Affiliations:** 1 Global Neuroscience Initiative Foundation, Los Angeles, CA, USA; 2 School of Health and Medical Sciences, Seton Hall University, South Orange, NJ, USA

**Keywords:** Traumatic brain injury, TDP-43, Taupathy, Dementia, Contact sports, Neurodegeneration, Concussion

## Abstract

Chronic traumatic encephalopathy (CTE) is a form of neurodegeneration that results from repetitive brain trauma. Not surprisingly, CTE has been linked to participation in contact sports such as boxing, hockey and American football. In American football getting "dinged" equates to moments of dizziness, confusion, or grogginess that can follow a blow to the head. There are approximately 100,000 to 300,000 concussive episodes occurring in the game of American football alone each year. It is believed that repetitive brain trauma, with or possibly without symptomatic concussion, sets off a cascade of events that result in neurodegenerative changes highlighted by accumulations of hyperphosphorylated tau and neuronal TAR DNA-binding protein-43 (TDP-43). Symptoms of CTE may begin years or decades later and include a progressive decline of memory, as well as depression, poor impulse control, suicidal behavior, and, eventually, dementia similar to Alzheimer's disease. In some individuals, CTE is also associated with motor neuron disease similar to amyotrophic lateral sclerosis. Given the millions of athletes participating in contact sports that involve repetitive brain trauma, CTE represents an important public health issue.

In this review, we discuss recent advances in understanding the etiology of CTE. It is now known that those instances of mild concussion or "dings" that we may have previously not noticed could very well be causing progressive neurodegenerative damage to a player's brain. In the future, focused and intensive study of the risk factors could potentially uncover methods to prevent and treat this disease.

## Introduction

Chronic traumatic encephalopathy (CTE) is a neurodegenerative disease that is believed to result from repetitive brain trauma. Not surprisingly, CTE has been linked to participation in popular contact sports such as boxing, American football, wrestling, and soccer. Characterized by a progressive taupathy and also marked by TAR DNA-binding protein-43 (TDP-43) proteinopathy, CTE has only been found in individuals with a history of repeated brain trauma (Gavett et al. 2011; McKee et al. 2009; McKee et al. 2010; Stern et al. 2011). Risk of brain trauma ranges from asymptomatic subconcussive blows to symptomatic concussion to more moderate or severe forms of traumatic brain injury (TBI). Originally termed "dementia pugilistica" because of its association with boxing, the neuropathology of CTE was first described by Corsellis in 1973 in a case series of 15 retired boxers (Corsellis et al. 1973). CTE results in a progressive decline of memory and cognition, as well as depression, suicidal behavior, poor impulse control, aggressiveness, and eventually dementia similar to Alzheimer's disease (AD). In some individuals CTE is associated with motor neuron disease, which appears clinically similar to amyotrophic lateral sclerosis (ALS). Given the millions of individuals, both young and old, participating in contact sports that involve repetitive brain trauma, as well as military personnel with repeated episodes of explosive blast-induced mild TBI (Elder et al. 2010), CTE represents an important public health issue.

This article will discuss recent advances in understanding the incidence, clinical expression, neuropathology (gross and microscopic), and management of CTE, with particular emphasis on contact sports-related CTE because it has been more consistently described in the medical literature.

## Epidemiology

In the United States, approximately 75% of individuals with TBI sustain a mild TBI also known as a concussion (Centers for Disease Control and Prevention: Report to Congress on mild traumatic brain injury in the United States: steps to prevent a serious public health problem. In. Atlanta (GA) 2003). According to the Consensus Statement on Concussion in Sport (McCrory et al. 2009), a concussion is defined as "a complex pathophysiological process affecting the brain, induced by traumatic biomechanical forces." For example, a direct blow to the head or elsewhere on the body with an impulsive force transmitted to the head may result in a graded set of neurological symptoms with or without loss of consciousness. Although concussion may result in neuropathological changes, the acute clinical symptoms largely result from cytoskeletal and metabolic disturbances that are temporary. No abnormality on standard structural neuroimaging studies is seen in concussion Moreover, the majority of deficits associated with a concussive injury are short-lived and typically resolve spontaneously within a matter of days, weeks, or months; however, it is important to note, that in a small percentage of individuals (~15%), post-concussion symptoms may be prolonged after the initial injury (McCrory et al. 2009). Concussed individuals sometimes experience the long-term effects of postconcussion syndrome, for months or even years, which can result in significant physical and emotional stress.

Contact sports athletes are commonly exposed to concussions. In 1928, Harrison Martland, a New Jersey pathologist and medical examiner, described a series of symptoms in boxers, which he termed "punch drunk," that appeared to result from the repeated blows to the head experienced in the sport (Martland HS: Punch-drunk. JAMA 1928). "Nearly one half of the fighters who have stayed in the game long enough," were described to exhibit cognitive, behavioral, or motor abnormalities that were well known to lay persons within the boxing community and referred to as "punch-drunk syndrome." In 1937, the more formal term *dementia pugilistica *was introduced (Millspaugh 1937), emphasizing the severity of cognitive dysfunction in boxers. Boxers present with various symptoms indicative of damage to the pyramidal and extrapyramidal systems which manifest most often as disturbed gait and coordination, slurred speech, and tremors, as well as cerebral dysfunction causing cognitive impairments and neurobehavioral disturbances including suicide (Omalu et al. 2010a). By 1973, dementia pugilistica was replaced by the term CTE, a neuropathologically distinct disorder due to repetitive head trauma not only in boxers (McCrory et al. 2007) but also in other contact sports (Corsellis et al. 1973).

Costanza and colleagues described the clinical progression of a former professional boxer who developed CTE (Costanza et al. 2011). The patient started boxing at 17 years of age and at 22 he was at the European championship level. At the age of 24, he began to exhibit bizarre behavior and from the age of 25, he developed a progressive symptomatology characterized by extra-pyramidal hyperkinetic-rigid syndrome, pyramidal signs, epilepsy, cognitive impairment, and psychiatric/behavioral symptoms. Neuropathological analysis after he died at the age of 58 of a pulmonary embolism revealed large numbers of neurofibrillary tangles (NFTs) concentrated in the supragranular layers of the neocortex (Costanza et al. 2011). Interestingly, the distribution of NFTs in neocortical association areas is unique to CTE and not observed in typical AD cases. NFTs in CTE were primarily located in superficial layers (II and upper III), whereas in AD they predominate in deep layers (V and VI), correlating with the location of neurons forming specific corticocortical connections (Costanza et al. 2011).

The frequency of CTE in professional boxers is estimated to be 0.8 brain injuries per 10 rounds (Heilbronner et al. 2009). Approximately 20% of retired professional boxers developed CTE (Jordan 2000; McCrory et al. 2007). Risk factors associated with CTE include increased exposure (e.g., duration of career, age of retirement, total number of bouts), poor performance, and increased sparring (Jordan 2000; Jordon et al. 1997). High-exposure boxers (12 or more professional bouts) had significantly higher measures on the Chronic Brain Injury (CBI) scale than low-exposure boxers, indicating that neurologic impairment is related to boxing exposure. Both amateur and professional boxers are potentially at risk of developing CTE although whether amateur boxing leads to chronic TBI has been disputed (Loosemore et al. 2007; Porter 2003; Haglund& Eriksson 1993). In a small, controlled, prospective study of competitive amateur boxers in Ireland, there was no evidence of a decline in cognitive function over nine years per raw scores and changes in score in several neuropsychological tests including the Trail Making Test Parts A and B and Digit Symbol (Porter 2003). The Trail Making Test is a test of visual attention and task switching. The Digit Symbol Test measures processing speed requiring the subject to copy symbols that match the numbers 1-9 according to a key. Both tests have been validated for assessing dementia (Ashendorf et al. 2008; Tierney et al. 2010).

Haglund and Eriksson (Haglund & Eriksson 1993) found a higher incidence of slight or moderate electroencephalography deviations and inferior finger-tapping performance among amateur boxers compared with two control groups of soccer players and track and field athletes, but no significant differences between the groups on computed tomography (CT) images or magnetic resonance imaging (MRI). In addition, there were no significant differences in the width of the ventricular system, anterior horn index, width of cortical sulci, signs of vermian atrophy, or the occurrence of a cavum septum pellucidum between Swedish amateur boxers and controls in a retrospective study with CT and MRI (Haglund & Bergstrand 1990).

Although initially seen in professional boxers, CTE has now been identified in a number of athletes competing in different contact sports including amateur and professional wrestling, professional hockey, professional soccer and American football. Professional wrestling is a contact sport with a high risk for players to sustain repeated concussions over their careers. Omalu and colleagues reported tissue substrates and forensic evidence for CTE in a professional American wrestler (Omalu et al. 2010b). Histochemical analysis revealed diffuse, sparse to frequent tau-immunoreactive NFTs in the neocortex, subcortical ganglia, and brainstem nuclei. Recently, Omalu and colleagues (Omalu et al. 2010c) presented a case of CTE in a retired National Football League (NFL) player. The brain tissue revealed diffuse cerebral taupathy (NFTs and neuritic threads (NT)) without any neuriticamyloidopathy. Omalu and colleagues reported the first two cases of CTE in retired NFL players in 2005 (Omalu et al. 2005) and 2006 (Omalu et al. 2006). Both patients' medical history included symptoms of cognitive impairment, a mood disorder, and parkinsonian symptoms after retirement. There was no family history of AD or any other head trauma outside of football. In the first case, a comprehensive neuropathological examination was performed approximately 12 years after retirement. On autopsy, the brain demonstrated no cortical atrophy, cortical contusion, or infarcts. There was mild neuronal cell loss in the frontal, parietal, and temporal neocortex. CTE was evident by the demonstration of NFTs or neuropil threads in the hippocampus or entorhinal cortex. (Omalu et al. 2005).

The second reported case of autopsy-confirmed CTE in a retired professional football player displayed neuropathological features that differed from those of the first reported case (Omalu et al. 2006). Relevant history included a 14-year span of play in organized football starting at the age of 18 years. The former athlete was diagnosed with major depressive disorder without psychotic features after retirement and after several failed attempts, committed suicide. Both cases of CTE exhibited tau positive NFTs and neuropil threads in the brain; however, amyloid plaques were completely absent in the second case (Omalu et al. 2005; Omalu et al. 2006). Reasons for the contrasting features in these two cases are not clear. More recently, 14 of the 15 professional American football players examined neuropathologically at the Veterans Affairs Center for the Study of Traumatic Encephalopathy (CSTE) Brain Bank have been diagnosed with CTE (Stern et al. 2011).

Cincinnati Bengals receiver Chris Henry (age 26) suffered from CTE before he died tragically in December 2009. Co-directors of the Brain Injury Research Institute at West Virginia, Julian Bailes, neurosurgeon, and BennetOmalu, California medical examiner, made the stunning announcement in early 2010 (Los Angeles Times Staff and Wire Reports: Bengals' Henry had chronic brain injury. In: Los Angeles Times. Los Angeles 2010). Henry was only 26 years old at the time of his death, yet his brain already showed the pathophysiological effects of CTE caused most likely by repetitive blows to the brain.

Because repetitive closed head injuries seem to be the cause of CTE, athletes involved in collision sports seem to be at a high risk of developing the disorder. All confirmed cases of CTE have had a history of progressive brain trauma. In American football, a "ding" is a concussion or mild TBI caused by a blow to the head or body. There are approximately 100,000 to 300,000 concussions occurring in the game of football alone each year (Guskiewicz et al. 2003). It is known that football players and boxers experience thousands of subconcussive blows during a career (Guskiewicz et al. 2003). Athletes at certain positions (e.g., linemen) may sustain up to 1,400 impacts per season, and high school players who play both offense and defense potentially sustain closer to 2,000 impacts (McCrory et al. 2009; Herring et al. 2006; Talavage et al. 2010; Crisco et al. 2010; Greenwald et al. 2008).

Recently, data based on direct measurements of head impact exposure in college football players demonstrated that running backs and quarterbacks suffer the hardest and most severe blows to the head, while linemen and linebackers suffer more head impacts during a game than players in other positions (Crisco et al. 2011). Nearly 300,000 head impacts at three institutions were recorded over three seasons. The researchers measured impacts with standard football helmets outfitted with sensors that recorded such data as head acceleration and impact location which allowed them to quantify the severity and frequency of blows to the head by player position. Offensive line and quarterbacks had the highest and lowest frequency, respectively, of front impacts. In contrast, quarterbacks received the greatest magnitude and the most frequent impacts to the back of the helmet. Data also suggest that top-of-head impacts have a higher risk of concussion than impacts to the front, right, or back of the head (Guskiewicz et al. 2007). Top-of-helmet impacts might result in causing the cerebellum to impact the base of the skull and recoil superiorly into the cerebellar tentorium.

It is without question that NFL players are at a heightened risk for cognitive impairment and dementia (Guskiewicz et al. 2005). In fact, since its inception in 2006, the 88 Plan, jointly run by the NFL and the NFL Players Association (NFLPA), has spent about $9.7 million toward the care of 132 former NFL players with dementia. The plan was named after former Baltimore Colts tight end John Mackey who wore the #88 jersey. Recently, the NFL and NFLPA expanded the 88 Plan to include coverage of qualifying players for reimbursement of expenses associated with ALS. Nevertheless, an athlete in any sport who may have sustained more than one concussive injury may be at a risk for CTE including, but not limited to hockey, rugby, lacrosse, martial arts, horseback riding, parachuting, and downhill skiing. There is a rising incidence of TBI in hockey and bodychecking, thought by some to be a useful skill for winning games, is a major risk for concussion in this sport (Marchie&Cusimano MD: Bodychecking and concussions in ice hockey: should our youth pay the price? JAMC 2003). Concussions are rarely caused by being struck with a puck (Honey 1998). In fact, the link between bodychecking and concussions is analogous to the association between smoking and lung cancer (Hill 1965). CTE has also been found in epileptics, physical abuse victims, and a circus clown (McKee et al. 2009; Gavett et al. 2011; Roberts et al. 1990). More worrisome and less publicized are the increasing number of concussions among younger, even little league baseball players.

According to an analysis by the Centers for Disease Control (CDC) and Prevention, emergency department visits for sport-related TBI, including concussions, among children and adolescents increased by 60% during the last decade (2001 to 2009) (Prevention CfDCa: Nonfatal traumatic brain injuries related to sports and recreation activities among persons aged < 19 years- United States 2001). The analysis in CDC's Morbidity and Mortality Weekly Report said that football, basketball, bicycling, and soccer were the primary sports involved. In Canada, 10-12% of minor league ice hockey players 9-17 years old who are injured report a head injury, most commonly a concussion (Cole 2003). A review of the literature published between 1966 and 1997 revealed that youth aged 5-17 years-old experienced approximately 2.8 concussions per 1000 player-hours of ice hockey; the number per 1000 player-hours was about the same among high school players (Honey 1998). Among Canadian amateur ice hockey players over 18-years-old, the rate is 4.6-6.0 concussions per 1000 player-hours (Goodman et al. 2001). These numbers are alarming since the younger developing brain is at an even higher risk of injury and repeated concussions may lead to permanent learning disabilities and other neurological and psychiatric problems. Pre-adolescent youth with a TBI may never fully develop the social and cognitive skills characteristic of adults and may be more violent than those without such an injury (Leon-Carrion & Ramose 2003; Benz et al. 1999). While the most common cognitive sequelae of concussion in children appear similar for children and adults, the recovery profile and breadth of consequences in children remains largely unknown, as does the influence of pre-injury characteristics (e.g., gender) and injury details (e.g., magnitude and direction of impact) on long-term outcomes.

## Clinical presentation

Clinically, the diagnosis of CTE depends on the presence of progressively evolving neuropsychiatric symptoms attributable to repeated brain trauma that cannot be attributed to other pathological processes. Unfortunately, data on the clinical manifestations of CTE have only recently been accumulating via post-mortem medical record review and interviews of friends or family members of individuals with neuropathologically documented CTE.

The clinical symptoms of CTE are first evident by deteriorations in attention, concentration and memory, as well as disorientation and confusion, and occasionally accompanied by dizziness and headaches. With progressive deterioration, additional symptoms, such as poor judgment, and overt dementia, become manifest. Severe cases of CTE are accompanied by a progressive slowing of muscular movements, a staggered and propulsive gait, masked facies, impeded speech, tremors, vertigo, and deafness (Millspaugh 1937). According to Corsellis et al. (Corsellis et al. 1973) an individual with CTE may progress through three stages of the disease beginning with affective disturbances and psychotic symptoms. As the disease progresses to the second stage, the individual may suffer from social instability, erratic behavior, memory loss, and the initial signs of Parkinson's disease (McKee et al. 2009). The third stage consists of a progressive deterioration to dementia, which is often accompanied by speech and gait abnormalities, dysarthria, dysphagia, and ptosis (McKee et al. 2009). Of 51 neuropathologically confirmed cases of CTE of which 90% occurred in athletes (85% boxers, 11% football players), the first symptoms of the disease were noticed at age ranges from 25 to 76 years. One third were symptomatic at the time of retirement from the sport and half were symptomatic within 4 years of stopping play. The severity of the disorder depends on which clinical stage the individual is in (McKee et al. 2009) and appears to correlate with the length of time engaged in the sport and the number of traumatic injuries; however, where a single TBI can trigger the onset of CTE remains a matter of speculation (McKee et al. 2009).

Mood and behavioral symptoms associated with CTE are most concerning to family members and colleagues and include depression, emotional instability, suicidal thoughts, and problems with impulse control (Stern et al. 2011). McKee et al. (McKee et al. 2009) recently reviewed the clinical symptoms of CTE in 51 neuropathologically confirmed cases of the disease in athletes. In 30% of cases there was a prominent mood disturbance, usually depression (28%). Substance abuse and suicide are common in CTE (Omalu et al. 2011) (Omalu et al. 2010a; Omalu et al. 2010b; Omalu et al. 2010c). Omalu and colleagues (Omalu et al. 2010a) recently presented five cases of professional American contact sport athletes aged 36-50 years of age who committed suicide. While the brains appeared grossly normal at autopsy, immunohistochemical analysis revealed widespread cerebral taupathy without neuritic plaques. In a subsequent paper the authors (Omalu et al. 2011) presented the case of CTE in a 27 year old US Marine Corps Iraqi war veteran who was exposed to mortar blasts and improvised explosive device blasts less than 50 meters away. Following his second deployment, he developed a progressive history of cognitive impairment, impaired memory, behavioral and mood disorders, and alcohol abuse. He committed suicide approximately eight months after his honorable discharge. His brain at autopsy appeared grossly normal; however, immunohistochemical analysis revealed CTE changes accentuated in the depths of the frontal cortical sulci (Omalu et al. 2010a; Omalu et al. 2011). Cincinnati Bengals receiver Chris Henry had suffered from CTE before he died at the age of 26 years. Most recently, a brain autopsy of a popular, 21-year-old defensive end on the University of Pennsylvania football team who committed suicide revealed that he suffered from CTE, the same repetitive brain trauma-induced disease found in more than 20 deceased National Football League (NFL) players, two of whom also committed suicide (Schwarz A: Suicide reveals signs of a disease seen in N.F.L. In: The New York Times. New York 2010). Thus, the brain damage found in NFL veterans can afflict younger players who may sustain thousands of subconcussive collisions while the brain is developing. It is unclear if any of the neuropathologic changes of CTE observed in the brain of individuals in their teens are associated with behavioral or cognitive symptoms at that time. (Omalu 2011).

Symptoms of CTE are consistent with those expected from the neuropathological changes observed at autopsy. For example, impairments in cognition are associated with medial temporal and dorsolateral frontal degeneration, changes in mood with amygdala degeneration, and changes in behavior with amygdala and orbitofrontal degeneration. Interestingly, a recent study showed that overweight NFL players have significantly more decreases in blood flow in the prefrontal cortex and temporal pole, and greater cognitive impairment than those retired NFL players categorized as normal weight (Willeumier et al. 2012). Thus, it appears that both playing professional American football and being overweight result in additive risk factors for decreasing blood flow and cognition. Although the disease process likely starts at the time of injury, the initial signs of CTE do not typically become evident until decades after the end of exposure to repetitive brain injury. When symptoms of CTE begin, the onset is earlier than that of sporadic AD and usually earlier than that of frontotemporal dementia (FTD).

## Diagnostic features

The diagnosis of CTE relies on postmortem tissue analysis; therefore, forensic pathologists play an important role in the identification and epidemiology of CTE since the disease can only be definitively diagnosed by direct tissue examination. Although the majority of professional and collegiate athletes examined for CTE have in fact been found to have had the disease, this represents a biased sample in that families who suspect their loved ones may be impaired are more likely to agree to brain donation for research purposes.

### Gross pathology

**Figure 1:**
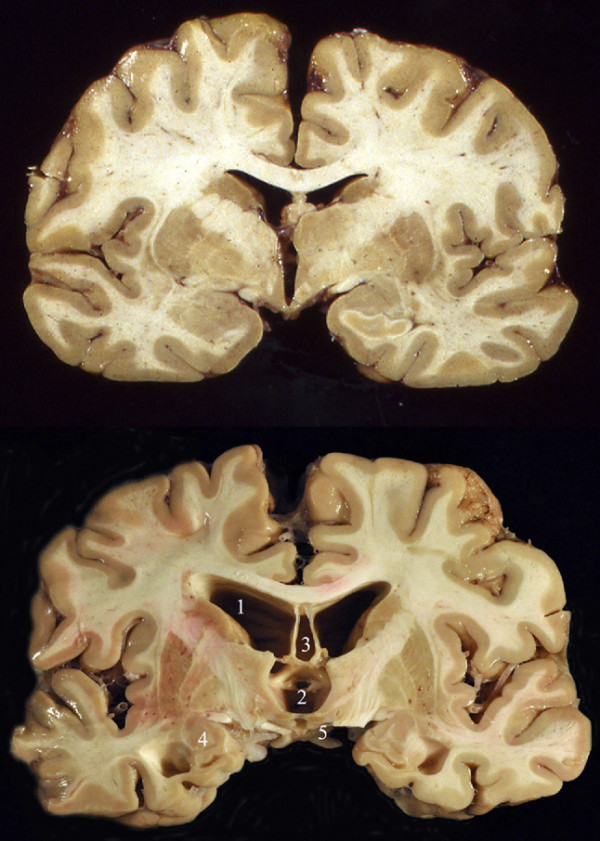
**Gross pathology of CTE**. **Top: **Coronal section of a normal human brain. **Bottom:** Coronal section of a brain from a retired professional football player, showing the characteristic gross pathology of CTE. Changes noted include severe dilatation of lateral ventricle (1) and third ventricle (2), cavum septum pellucidum (3), marked atrophy of the medial temporal lobe structures (4), and shrinkage of the mammillary bodies (5). Reprinted with permission from Stern et al. 2011 (Stern et al. 2011).

Postmortem tissue analysis of athletes with a history of repeated mild head injuries have produced several consistent findings that, together, make CTE a distinctive disorder. The gross neuropathologic changes of CTE, which are typically observed in late-stage disease, include generalized atrophy, most prominent in the frontal and medial temporal lobes; enlargement of the lateral and third ventricles; cavum septum pellucidum; and usually septal fenestrations (see Figure 1) (Stern et al. 2011). Additional gross changes include shrinkage of the mammillary bodies, and atrophy of the hippocampus, entorhinal cortex, and amygdala, which may result in an overall reduction in brain size (Gavett et al. 2011; McKee et al. 2009). Thinning of the hypothalamic floor and pallor of the locus coeruleus and substantianigra have also been noted (Gavett et al. 2011; McKee et al. 2009; Stern et al. 2011).

### Microscopic neuropathology

**Figure 2:**
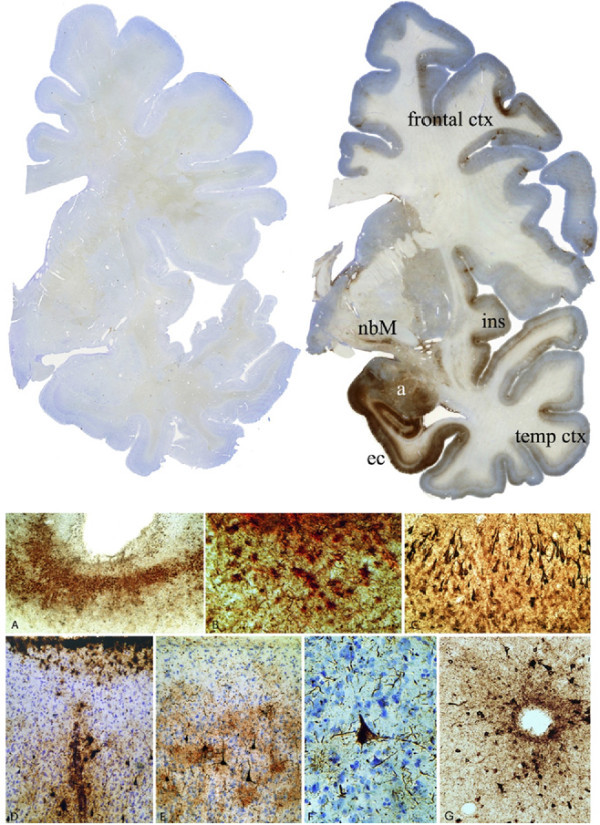
**Microscopic neuropathology of CTE**. **Top panel: **Phosphorylated tau (AT8) immunostained coronal hemisections of a normal brain (left) and a brain from a retired professional football player with CTE (right). The brain with CTE displays severe neurofibrillary degeneration of the amygdala (a), entorhinal cortex (ec), temporal cortex, insular cortex (ins), nucleus basalis of Meynert (nbM), and frontal cortex. The cortical changes are more pronounced at the depths of the sulci. **Lower panels: **(A) AT8-positive neurofibrillary tangles (NFTs) are often prominent at the depths of the cortical sulci (original magnification: ×60). (B) Subpial AT-8- immunoreactive tangles are found in both neurons and astrocytes (double-immunostained section for GFAP (red) and AT8 (brown), showing colocalization of tau and GFAP; ×350). (C) Dense AT8-NFTs are found in the medial temporal lobe, including the CA1 region of the hippocampus, shown here. (×150). (D) AT-8-positive NFTs and astrocytic tangles tend to be centered around small blood vessels and in subpial patches (×350). (E) NFTs characteristically involve cortical layers II and III (×150). (F) NFT in a Betz cell of the primary motor cortex (×350). (G) Perivascular tau-immunoreactive NFTs in CTE (×150). Reprinted with permission from Stern et al. 2011 (Stern et al. 2011).

Microscopically, CTE is characterized by a unique pattern of extensive tau-immunoreactive NFTs primarily distributed in the frontal and temporal cortex (see Figure 2). In advanced cases, tau-immunoreactive inclusions in the limbic and paralimbic regions, diencephalon, brainstem, and subcortical white matter have also been reported (Stern et al. 2011). The specific soluble and insoluble tau isoforms that are found in the NFTs in CTE are indistinguishable from NFTs in AD (Schmidt et al. 2001) and the ratio of tau isoforms with four versus three microtubule binding repeats is approximately 1 in both diseases (McKee et al. 2009). In the hippocampus and entorhinal cortex, the NFT distribution is comparable to that observed in AD, with numerous NFTs in the CA1 field, subiculum and layer II and V of the entorhinal cortex (Hof et al. 1992). However, β-amyloid deposition is an inconstant feature in CTE, in contrast to the extensive β-amyloid plaques that characterize nearly all cases of AD. Approximately 50% of CTE patients lack significant β-amyloid plaque accumulation (McKee et al. 2009). In addition, when β-amyloid deposits do occur they are less dense than in AD (McKee et al. 2009). Moreover, the tau immunoreactive abnormalities in CTE tend to cluster at the depths of sulci, around small blood vessels, and in superficial cortical layers (Gavett et al. 2011; McKee et al. 2009). Thus, neuropathologically, the pattern of the neurofibrillary abnormalities is distinct from AD especially when considering the predominance of tau pathology over β-amyloid accumulation in affected regions of the brain.

In addition to extensive tau-positive NFTs, the majority of CTE cases are also marked by widespread TDP-43 proteinopathy, that in some individuals is manifest as motor neuron disease (McKee et al. 2010; King et al. 2010). TDP-43 is a 414 amino acid nuclear protein encoded by the TARDBP gene on chromosome 1 (see (Bigio 2008; Wang et al. 2008) for reviews). TDP-43 is a highly conserved protein ubiquitously expressed in many tissues including the CNS where it is present in neuronal and glial nuclei and to a lesser extent in the cytoplasm.

**Figure 3:**
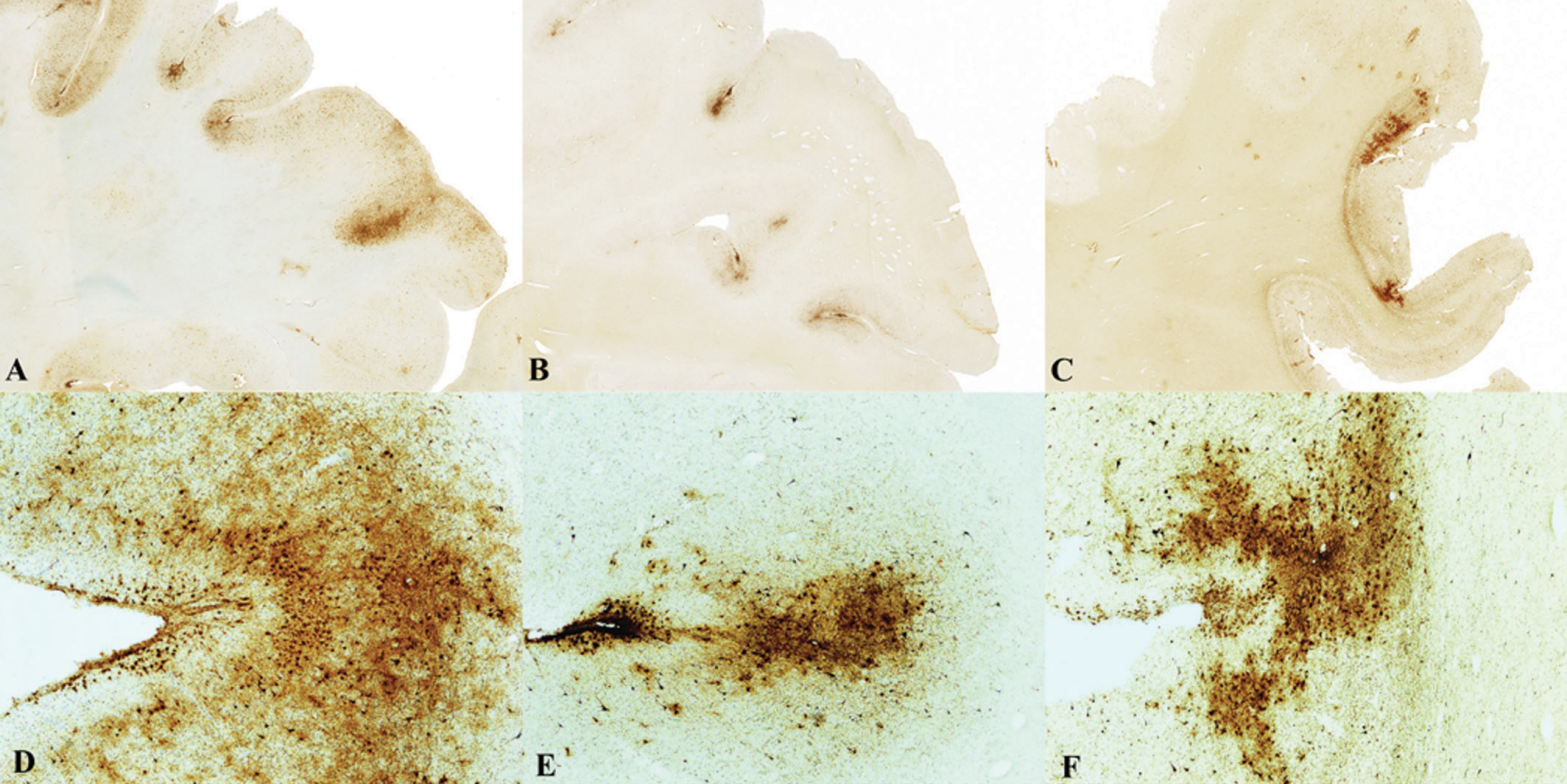
**Three cases of CTE with abundant TAR DNA-binding protein of approximately 43 kd (TDP-43) immunoreactivity and motor neuron disease**. (A-C) TDP-43 immunoreactive neurofibrillary degeneration in the frontal cortex of 3 cases of CTE + motor neuron disease (whole-mount 50 μm sections immunostained for CP13, original magnification: ×1) (D-F) Tau-positive neurofibrillary tangles, glial tangles, and neuropilneurites are particularly dense at the depth of the cortical sulci (×50). Adapted with permission from McKee et al. 2010 (McKee et al. 2010).

McKee and colleagues (McKee et al. 2010) examined 12 cases of CTE, and, in 10, found widespread TDP-43 immunoreactivity in the frontal and temporal cortex, medial temporal lobe, basal ganglia, diencephalon, and brainstem. The accumulation of TDP-43 as well as tau in the neocortex, medial temporal lobes, and deeper brain structures likely contributes to the overall clinical manifestations of cognitive and memory loss, behavioral changes, and parkinsonism (McKee et al. 2010). Three athletes with CTE also developed a progressive motor neuron disease that appears very similar to ALS (Brooks et al. 2000) and is characterized by profound weakness, atrophy, spasticity, and fasciculations several years before death. In these 3 cases there were abundant TDP-43-positive inclusions and neuritis in the spinal cord in addition to tau NFTs, motor neuron loss, and corticospinal tract degeneration (see Figure 3). Criteria for diagnosis of ALS include evidence of both lower and upper motor neuron degeneration and in terms of neuropathology, degeneration of motor neurons with ubiquitin-positive cytoplasmic inclusions and corticospinal tract degeneration (Hirano 1996). In addition, in sporadic ALS, remaining motor neurons often have ubiquitin-and TDP-43-immunoreactive inclusion bodies. This is the first pathological evidence that repeated brain trauma experienced in contact sports might be associated with the development of a motor neuron disease and suggests that some forms of clinical ALS may be associated with TBI.

Interestingly, TPD-43 inclusions are also found in frontotemporal lobar degeneration (FTLD), which shares a number of clinical and pathological features with ALS. Originally, TDP-43 was thought to be specific to ALS and FTLD, but it is now recognized to be present in a variety of disorders (Geser et al. 2010), indicating that these entities may share some pathogenic mechanisms related to the pathological expression of this protein in CTE. Although the physiological functions of TDP-43 are incompletely understood, by virtue of its capacity to bind to neurofilament mRNA and stabilize the mRNA transcript (Strong et al. 2007), TDP-43 plays a critical role in mediating the response of the neuronal cytoskeleton to axonal injury. Importantly, TPD-43 pathology develops very early in the disease state and precedes overt neurodegeneration by some as yet unspecified period of time. Other recent studies demonstrate the absence of TDP-43 proteinopathy in acute and long-term survivors of a single TBI in humans (Johnson et al. 2011). Multiple regions were examined including the hippocampus, medial temporal lobe, cingulate gyrus, superior frontal gyrus and brainstem. Thus, the absence of TDP-43 proteinopathy may indicate a fundamental difference in the processes following a single TBI from those of repetitive TBI (Johnson et al. 2011).

Omalu recently reported (Omalu 2011) the case of CTE in an 18 year old high school student who died suddenly and unexpectedly following repeated concussions while engaged in contact sports in school. Autopsy revealed microscopic findings consistent with CTE. What is very significant about this case is the evidence of restricted tauopathy observed at such a young age. The taupathy did not contribute to his sudden death; however, it was diagnostic of incipient CTE, which the authors concluded may have occurred had the individual survived into his third- fifth decades of life (Omalu 2011).

The biological mechanisms by which repetitive brain trauma may lead to CTE are not known. Recently, Blaylock and Maroon hypothesized that a process called immunoexcitotoxicity may play a major role in the development of progressive neurodegeneration in CTE (Blaylock & Maroon 2011). According to the hypothesis, chronic activation of microglia due to repetitive brain trauma causes high levels of glutamate and a significant accumulation of hyperphosphorylated tau protein resulting in the observed NFT accumulation in CTE. Compelling research has linked excess glutamate stimulation and/or elevations of proinflammatory cytokines to a number of neuropsychiatric and behavioral conditions, many of which are seen with CTE. Moreover, oxidative stress induced by glutathione depletion reproduces pathological modifications of TDP-43 linked to TDP-43 proteinopathies (Iguchi et al. 2011). More research is needed to develop a set of criteria that provide a reliable and valid indicator of neuropathologically verified CTE. Examining the role of immunoexcitotoxicity and oxidative stress in CTE may provide important early markers of the disease and information on how to strengthen the immune system to combat inflammation that could significantly minimize brain damage.

## Genetic risk and role of APOE ε4

Genetic disposition appears to be an important potential risk factor for CTE and may one day explain why some people develop CTE while most do not. One of the genes thought to influence CTE risk is the apolipoprotein E (APOE = gene, apoE = protein) gene. As the major lipoprotein involved in lipid transport and metabolism (Mahley 1988), apoE expression is implicated in neuronal growth and in repair after injury of both peripheral and central neurons (Snipes et al. 1986; Ignatius et al. 1986). There are three known isoforms of apoE, coded for by the APOE ε2, APOE ε3, and APOE ε4 genes. Based on genetic testing conducted in conjunction with neuropathologic examinations of individuals with a history of repeated head injuries, studies (Jordon et al. 1997; Teasdale et al. 1997; Kutner et al. 2000) have linked, specifically the APOE ε4 allele, to worse cognitive functioning in boxers and professional American football players (Samatovicz 2000). In 10 cases of CTE, 50% carried at least one APOE ε4 allele and 1 was homozygous for APOE ε4 (McKee et al. 2009). In addition, all boxers with severe impairment as measured on the CBI scale possessed at least one APOE ε4 allele (Jordon et al. 1997). When contrasted with the estimated 15% of the global population possessing at least one APOE ε4 allele (Singh et al. 2006; Hill et al. 2007), the frequency of this allele in those with CTE seems higher than expected. Thus, the inheritance of an APOE ε4 allele might be a risk factor for the development of CTE.

Epidemiologic data have also implicated the APOE ε4 genotype as a risk factor for the development of AD after TBI (Mayeux et al. 1995; Mayeux et al. 1993; Jordan 2000; Jordon et al. 1997; Jordon 2007; McCrory 2007). Acute TBI induces Aβ deposition in 30% of people and a significant proportion of these individuals are heterozygous for APOE ε4 (Nicoll et al. 1995). Carrying the APOE ε4 allele is a risk factor for early onset AD and is the only consistently identified risk factor for late-onset AD. Carriers of two APOE ε4 alleles have a higher risk and earlier onset of AD than heterozygous subsets (Saunders et al. 1993). Recent genetic association studies have explored the possible role of several other gene polymorphisms in the neuropathological changes of CTE; however, evidence for correlation with APOE ε4 is the strongest.

## Clinical diagnosis

The clinical diagnosis of CTE is difficult because presently there are no available biomarkers and no consensus diagnostic criteria. In addition, its clinical presentation can be similar to AD, FTD, sporadic ALS, and other neurodegenerative diseases. Thus, the differential diagnosis of CTE often includes these diseases especially in the later stages of the disorder. Older individuals with memory problems may seem to have AD, and, in fact, may have evidence of AD and CTE neuropathologically. When the age at onset is earlier and accompanied by changes in behavior, it may be difficult to rule out FTD (Gavett et al. 2011). Although a history of repetitive head trauma is suggestive of CTE, head trauma is also one of the strongest contenders for initiating the molecular events that result in other neurodegenerative processes such as AD (Fleminger et al. 2003; Plassman et al. 2000; Mortimer et al. 1985), FTD (Jawaid et al. 2009)and Parkinson disease (Goldman et al. 2006). In addition, the etiology of sporadic ALS has long been suspected to include a history of trauma to the brain and spinal cord (Strickland et al. 1996; Chen et al. 2007), a history of participation in athletics (Scarmeas et al. 2002), and strenuous physical activity (Scarmeas et al. 2002; Longstreth et al. 1998; Chiò et al. 2005; Belli &Vanacore 2005). Thus, without neuropathologic confirmation, a diagnosis of CTE cannot be made with confidence.

The overlap between CTE and AD involves not only the similarities in the clinical expression of these disorders but also their pathogenesis and epidemiology. Pathologically, CTE shares several characteristics of AD including abundant, widespread NFTs, acetylcholine deficiency, and hyperphosphorylated tau (Hof et al. 1992; McKee et al. 2009). However, there are some important differences. Clinically, CTE typically presents with age of onset in the 40s and 50s as opposed to onset after 65 years in sporadic AD. In addition, the clinical progression of the disease is much slower, often lasting decades, and is characterized by a subtle deterioration in personality and behavior (Daneshvar et al. 2011). Neuropathologically, the pattern of the neurofibrillary abnormalities is distinct from AD especially when considering the predominance of tau pathology over β-amyloid accumulation in affected regions of the brain.

From an epidemiological viewpoint, both positive and negative data were reported in respect to the association between a history of previous head trauma and risk of AD. Meta-analyses supported a significant association between head injury and AD, but only in men (Fleminger et al. 2003; Mortimer et al. 1985; Gavett et al. 2010). How brain injury can trigger the neurodegenerative changes resulting in AD is still controversial. In addition, AD-like neurodegenerative changes following brain injury may be present alone or in conjunction with other types of neurodegenerative lesions.

The similarities between CTE and FTD focus on TDP-43 proteinopathy. TDP-43 was recently established as the major pathological protein in FTD with ubiquitin-positive and tau-negative inclusions, with or without motor neuron disease/ALS, and in sporadic ALS, establishing the concept of a spectrum of "TDP-43 proteinopathies." This protein is also widely present in CTE and in some cases the TDP-43 proteinopathy involves the spinal cord and may be associated with ALS (McKee et al. 2010).

In recent years research to investigate other neurodegenerative diseases, like AD, has been moving to integrate clinical (e.g., neuropsychological tests), biological (e.g., cerebrospinal fluid), anatomical (e.g., neuroimaging), and genetic information (e.g., APOE genotype) for the purposes of differential diagnosis, prevention, and treatment. This type of research approach is clearly needed in order to arrive at the timely diagnosis of CTE.

## Clinical guidelines for management

CTE is the only known neurodegenerative disease with a specific identifiable cause; in this case, repetitive head trauma, with or without symptomatic concussion. Clearly, the easiest way to decrease the incidence of CTE in athletes is to decrease the incidence of repetitive brain trauma at all levels of play. Nevertheless, in the US, approximately 1.7 million sports-and recreation-related TBIs occur each year. About 75% of these cases are classified as mild TBIs or concussions (Daneshvar et al. 2011). In addition, players in collision sports such as American football may experience many more subconcussive impacts throughout a season and career.

Over the last 10 years, as part of the Heads Up initiative (in Youth Sports & Activity 2007), the CDC has worked to raise awareness about concussions, and improve prevention, recognition, and response to this injury among health care professionals, parents, coaches, children and adolescents. Nevertheless, despite the increased attention given to the frequent occurrence of sports-related concussion, effective measures of prevention of CTE in all levels of athletic involvement are still lacking. Several early "return to play" (RTP) guidelines for athletes who have sustained concussions have been proposed including the Cantu guidelines (Cantu 1986), the Colorado guidelines (Colorado Medical Society 1991), and the American Academy of Neurology guidelines for concussion management (American Academy of Neurology 1997). Current RTP guidelines recommend a graduated increase in the level of the activity of the athlete progressing from the initial stage of "light exercise" towards "full contact" activity once the athlete is completely symptom-free at rest. The progression may be complete in as little as five days or may take as long as the athlete requires. According to Robert Stern, co-director of CSTE, new evidence shows that 85% of concussions require about three weeks of recovery (Abel D: Bill addressing student concussions advances. In: Boston Globe 2010). Studies using event-related potentials, transcranial magnetic stimulation, balance testing, multitask effect on gait stability, PET and diffusion tensor MRI (DTI) have all shown abnormalities in concussed athletes or non-athletes with TBI lasting for 2 to 4 weeks (De Beaumont et al. 2007; Arfanakis et al. 2002; Gosselin et al. 2006). Thus, safe RTP guidelines might require at least 4 to 6 weeks to facilitate complete recovery and to protect from re-injury, as a second concussion occurs much more frequently in the immediate period after a concussion (Guskiewicz et al. 2003; Mayers 2008). Returning an athlete too early to sport after a concussion may place the athlete at risk for second-impact syndrome, in which concussed cells may be irreversibly damaged by the occurrence of swelling if a second concussion is sustained (Signoretti et al. 2011). The largest number of sudden deaths (57%) in young, competitive athletes due to second-impact syndrome was in football (Thomas et al. 2011). Stern offers his own recommendations specifically for the sport of football including reducing full-contact practices, changing the way players line up on the field, and using new helmets to reduce the force of the impact on the football players' brains (Abel D: Bill addressing student concussions advances. In: Boston Globe 2010). The best recommendations for the prevention of CTE is to ensure that athletes who sustain concussions be seen by sports medicine professionals who have experience in treating concussions. Parents need to also be educated as to the signs and symptoms of concussion as they may be in the best situation to detect subtle changes in their child's behavior that others may not notice.

The specific nature of the brain trauma exposure also needs to be carefully studied. It is unknown whether CTE is more likely to occur after extended exposure to repetitive brain trauma or whether a single concussion can initiate the neurodegenerative cascade in susceptible individuals. In addition, all neuropathologically confirmed cases of CTE have had a history of repeated head trauma; however, not all individuals with exposure to brain trauma develop CTE. Thus, the contribution of other variables, such as age, gender, and genetic predisposition also require further investigation, as does the role of oxidative stress in the disease process.

A major goal of research must be epidemiologic and prospective studies to identify the specific risk factors for the development of this neurodegenerative disease. Per the CSTE, a large study is underway involving a number of former NFL players to study their lives and then examine their brains following death. To this end, more than 500 living athletes have agreed to donate their brain and spinal cord to the CSTE upon death, including over a dozen former hockey players (Boston University Researchers Report 2011). The VA CSTE Brain Donation Registry allows for current and former athletes and military personnel to pledge to donate their brain and spinal cord to the CSTE (Brain Donation Registry).

## Conclusion

CTE is a neuropathologically distinct neurodegenerative disease that is believed to result from repetitive brain trauma as frequently occurs in popular contact sports. Early symptoms of CTE include a decline of memory and cognitive functioning, depression, poor impulse control and in many cases suicide. Disease progression is slow and eventually leads to dementia. In some individuals CTE may also lead to a motor neuron disease similar to ALS. What was once believed to occur only in boxers is now openly discussed as a potential consequence of repetitive brain trauma seen in multiple different sports and at all levels of play. Although we now know that repetitive brain trauma sets off a cascade of molecular events that results in neurodegeneration marked by a unique taupathy and TDP-43 proteinopathy, we do not know the underlying mechanism of the disease. How can CBT be detected and diagnosed accurately during life? How can CBT be prevented and treated? By addressing these important questions, CBT can move from a disease only diagnosed postmortem to one that can be identified, treated, and cured in life. In addition, new research will provide policy makers with scientific data to make appropriate guidelines regarding the treatment of brain trauma in athletes and military personnel. Given the millions of athletes both young and old participating in contact sports that involve repetitive brain trauma, CTE remains an important and expanding health issue with unmet medical need.

## Abbreviations

APOE: apolipoprotein E gene; apoE: apolipoprotein E protein; AD: Alzheimer's disease; ALS: amyotrophic lateral sclerosis; CDC: Center for Disease Control; CSTE: Center for the Study of Traumatic Encephalopathy; CT: computerized tomography; CTE: chronic traumatic encephalopathy; FTD: frontotemporal degeneration; FTLD: frontotemporal lobar degeneration; MRI: magnetic resonance imaging; NFL: National Football League; NFLPA: NFL Players Association; NFT: neurofibrillary tangle; NT: neuritic threads; RTP: return to play; TBI: traumatic brain injury; TDP-43: TAR DNA-binding protein-43

## Competing interests

The authors declare that they have no competing interests.

## Authors' contributions

All authors participated in the preparation of the manuscript, and read and approved the final manuscript.
